# Supplementary material for: Empirical methods for controlling false positives and estimating confidence in ChIP-Seq peaks
Source: BMC Bioinformatics. 2008 Dec 5;9:523. doi: 10.1186/1471-2105-9-523 (PMC2628906; doi:10.1186/1471-2105-9-523)
Supplement: Additional File 1 — A variety of html documents from the USeq web site detailing the available applications, their best usage, output file type descriptions, command line menus, etc. [file 1471-2105-9-523-S1.zip › USeqUserGuides/JavaDoc.zip!/JavaDoc/bioroot/parsers/ExtractWords.html]

ExtractWords


|  |  |  |
| --- | --- | --- |
| **Overview** \| **Package** \| **Class** \| **Use** \| **Tree** \| **Deprecated** \| **Index** \| **Help** | |  |
| PREV CLASS   **NEXT CLASS** | **FRAMES**    **NO FRAMES**     **All Classes** |  |
| SUMMARY: NESTED \| FIELD \| CONSTR \| METHOD | DETAIL: FIELD \| CONSTR \| METHOD |  |


---


## bioroot.parsers Class ExtractWords

```
java.lang.Object
  bioroot.parsers.ExtractWords
```

---

``` public class ExtractWords extends java.lang.Object ```

**Author:**
:   nix

---

**Constructor Summary**

`ExtractWords(java.lang.String[] args)`


| **Method Summary** | |
| --- | --- |
| `static java.lang.String[]` | `loadFile(java.io.File file)`             Loads a file's lines into a String[], will save blank lines. |
| `static java.util.HashSet` | `loadFileIntoHashSet(java.io.File file)`             Loads a file's lines into a hash set, keys only. |
| `static void` | `main(java.lang.String[] args)` |

**Methods inherited from class java.lang.Object**

`equals, getClass, hashCode, notify, notifyAll, toString, wait, wait, wait`

**Constructor Detail**

### ExtractWords

```
public ExtractWords(java.lang.String[] args)
```


**Method Detail**

### loadFile

```
public static java.lang.String[] loadFile(java.io.File file)
```

:   Loads a file's lines into a String[], will save blank lines.

---


### loadFileIntoHashSet

```
public static java.util.HashSet loadFileIntoHashSet(java.io.File file)
```

:   Loads a file's lines into a hash set, keys only.

---


### main

```
public static void main(java.lang.String[] args)
```


---


|  |  |  |
| --- | --- | --- |
| **Overview** \| **Package** \| **Class** \| **Use** \| **Tree** \| **Deprecated** \| **Index** \| **Help** | |  |
| PREV CLASS   **NEXT CLASS** | **FRAMES**    **NO FRAMES**     **All Classes** |  |
| SUMMARY: NESTED \| FIELD \| CONSTR \| METHOD | DETAIL: FIELD \| CONSTR \| METHOD |  |


---
